# Florabank1: a grid-based database on vascular plant distribution in the northern part of Belgium (Flanders and the Brussels Capital region)

**DOI:** 10.3897/phytokeys.12.2849

**Published:** 2012-05-16

**Authors:** Wouter Van Landuyt, Leo Vanhecke, Dimitri Brosens

**Affiliations:** 1 Research Institute for Nature and Forest, Kliniekstraat 25, 1070, Brussels, Belgium; 2 Flo.Wer, Bouchout Domain, Nieuwelaan 38, 1070, Meise, Belgium; 3 NBGB (National Botanic Garden of Belgium), Bouchout Domain, Nieuwelaan 38, 1860, Meise, Belgium

**Keywords:** Tracheophyta, grid mapping, flora, indigenous species, archeophytes, naturalised aliens

## Abstract

Florabank1 is a database that contains distributional data on the wild flora (indigenous species, archeophytes and naturalised aliens) of Flanders and the Brussels Capital Region. It holds about 3 million records of vascular plants, dating from 1800 till present. Furthermore, it includes ecological data on vascular plant species, redlist category information, Ellenberg values, legal status, global distribution, seed bank etc. The database is an initiative of “Flo.Wer” (www.plantenwerkgroep.be), the Research Institute for Nature and Forest (INBO: www.inbo.be) and the National Botanic Garden of Belgium (www.br.fgov.be). Florabank aims at centralizing botanical distribution data gathered by both professional and amateur botanists and to make these data available to the benefit of nature conservation, policy and scientific research.

The occurrence data contained in Florabank1 are extracted from checklists, literature and herbarium specimen information. Of survey lists, the locality name (verbatimLocality), species name, observation date and IFBL square code, the grid system used for plant mapping in Belgium (Van Rompaey 1943), is recorded. For records dating from the period 1972–2004 all pertinent botanical journals dealing with Belgian flora were systematically screened. Analysis of herbarium specimens in the collection of the National Botanic Garden of Belgium, the University of Ghent and the University of Liège provided interesting distribution knowledge concerning rare species, this information is also included in Florabank1. The data recorded before 1972 is available through the Belgian GBIF node (http://data.gbif.org/datasets/resource/10969/), not through FLORABANK1, to avoid duplication of information. A dedicated portal providing access to all published Belgian IFBL records at this moment is available at: http://projects.biodiversity.be/ifbl

All data in Florabank1 is georeferenced. Every record holds the decimal centroid coordinates of the IFBL square containing the observation. The uncertainty radius is the smallest circle possible covering the whole IFBL square, which can measure 1 Km² or 4 Km². Florabank is a work in progress and new occurrences are added as they become available; the dataset will be updated through GBIF on a regularly base.

## Data published through

GBIF: http://ipt.inbo.be/resource.do?r=florabank1

### Taxonomic coverage

Note: The taxonomic reference for the florabank1 database is the 1998 edition of the Belgian Flora by Lambinon et al. 1998.

### General taxonomic coverage description

**Figure 1.**
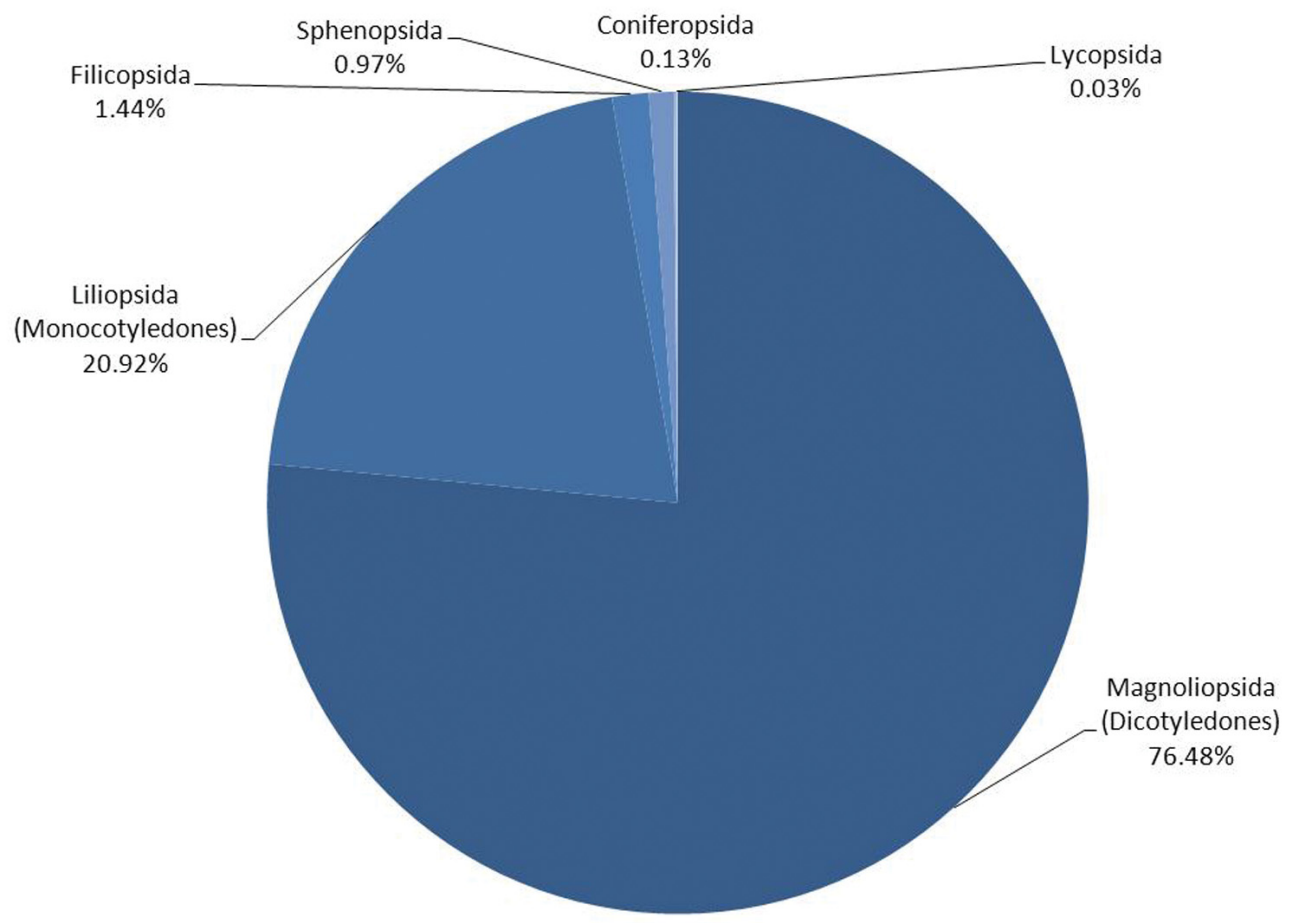
Taxonomic coverage of Florabank1

The coverage (figure 1) of this database spans the Phylum Tracheophyta or vascular plants. The highest number of records are from the Magnoliopsida (76.48%) followed by Monocotyledones (20.92%), Filicopsida (1.44%), Sphenopsida (0.97%) Coniferopsida (0.13%) and Lycopsida (0.03%). Ginkgopsids are within the scope of Florabank, but do not occur within the geographical scope of the database.

## Taxonomic ranks

**Phylum:** Tracheophyta

**Class:** Coniferopsida, Filicopsida, Ginkgopsida, Liliopsida (Monocotyledones), Lycopsida, Magnoliopsida (Dicotyledones), Sphenopsida

**Order:** Pinales, Taxales, Filicales, Marsileales, Ophioglossales, Osmundales, Salviniales, Ginkgoales, Alismatales, Arales, Commelinales, Cyperales, Hydrocharitales, Juncales, Liliales, Najadales, Orchidales, Poales, Pontederiales, Typhales, Typhales, Zingiberales, Isoetales, Lycopodiales, Selaginellales, Apiales, Apiales, Aristolochiales, Asterales, Callitrichales, Campanulales, Capparales, Caryophyllales, Celastrales, Cornales, Cucurbitales, Dipsacales, Elaeagnales, Ericales, Euphorbiales, Fabales, Gentianales, Geraniales, Haloragales, Haloragales, Hamamelidales, Juglandales, Lamiales, Linales, Loganiales, Magnoliales, Malvales, Myricales, Myrtales, Nymphaeales, Oleales, Paeoniales, Papaverales, Piperales, Plantaginales, Plumbaginales, Polemoniales, Polygalales, Polygonales, Primulales, Ranunculales, Rhamnales, Rosales, Rubiales, Rutales, Salicales, Santalales, Sapindales, Sarraceniales, Saxifragales, Scrophulariales, Theales, Thymelaeales, Urticales, Violales, Violales, Gymnospermae, Equisetales

**Family:** Araucariaceae, Cupressaceae, Pinaceae, Taxodiaceae, Taxaceae, Adiantaceae, Aspleniaceae, Blechnaceae, Dennstaedtiaceae, Dryopteridaceae, Hymenophyllaceae, Polypodiaceae, Thelypteridaceae, Woodsiaceae, Marsileaceae, Ophioglossaceae, Osmundaceae, Azollaceae, Salviniaceae, Ginkgoaceae, Alismataceae, Butomaceae, Araceae, Lemnaceae, Commelinaceae, Cyperaceae, Hydrocharitaceae, Juncaceae, Agavaceae, Alliaceae, Amaryllidaceae, Dioscoreaceae, Iridaceae, Liliaceae, Aponogetonaceae, Juncaginaceae, Najadaceae, Potamogetonaceae, Ruppiaceae, Scheuchzeriaceae, Zannichelliaceae, Zosteraceae, Orchidaceae, Poaceae, Pontederiaceae, Sparganiaceae, Typhaceae, Cannaceae, Isoetaceae, Lycopodiaceae, Selaginellaceae, Apiaceae, Araliaceae, Aristolochiaceae, Asteraceae, Callitrichaceae, Campanulaceae, Lobeliaceae, Brassicaceae, Capparaceae, Resedaceae, Aizoaceae, Amaranthaceae, Caryophyllaceae, Chenopodiaceae, Molluginaceae, Nyctaginaceae, Phytolaccaceae, Portulacaceae, Aquifoliaceae, Celastraceae, Cornaceae, Cucurbitaceae, Adoxaceae, Caprifoliaceae, Dipsacaceae, Valerianaceae, Elaeagnaceae, Actinidiaceae, Clethraceae, Empetraceae, Ericaceae, Monotropaceae, Pyrolaceae, Buxaceae, Euphorbiaceae, Caesalpiniaceae, Fabaceae, Betulaceae, Fagaceae, Apocynaceae, Asclepiadaceae, Gentianaceae, Balsaminaceae, Geraniaceae, Limnanthaceae, Oxalidaceae, Tropaeolaceae, Gunneraceae, Haloragaceae, Hippuridaceae, Hamamelidaceae, Platanaceae, Juglandaceae, Boraginaceae, Lamiaceae, Verbenaceae, Linaceae, Buddlejaceae, Calycanthaceae, Magnoliaceae, Malvaceae, Tiliaceae, Myricaceae, Lythraceae, Onagraceae, Trapaceae, Cabombaceae, Ceratophyllaceae, Nymphaeaceae, Oleaceae, Paeoniaceae, Fumariaceae, Papaveraceae, Saururaceae, Plantaginaceae, Plumbaginaceae, Convolvulaceae, Cuscutaceae, Hydrophyllaceae, Menyanthaceae, Polemoniaceae, Solanaceae, Polygalaceae, Polygonaceae, Primulaceae, Berberidaceae, Menispermaceae, Ranunculaceae, Rhamnaceae, Vitaceae, Amygdalaceae, Malaceae, Rosaceae, Rubiaceae, Anacardiaceae, Rutaceae, Simaroubaceae, Zygophyllaceae, Salicaceae, Loranthaceae, Santalaceae, Aceraceae, Hippocastanaceae, Sapindaceae, Staphyleaceae, Droseraceae, Crassulaceae, Grossulariaceae, Hydrangeaceae, Saxifragaceae, Bignoniaceae, Globulariaceae, Lentibulariaceae, Martyniaceae, Orobanchaceae, Pedaliaceae, Scrophulariaceae, Elatinaceae, Hypericaceae, Thymelaeaceae, Cannabaceae, Moraceae, Ulmaceae, Urticaceae, Begoniaceae, Cistaceae, Frankeniaceae, Loasaceae, Passifloraceae, Tamaricaceae, Violaceae, Ephedraceae, Equisetaceae

**Common names:** conifers, ferns, ginkgos, monocots, lycopods, dicots, horsetails

## Spatial coverage

**General spatial coverage:** Florabank deals with distribution data of the wild flora of Flanders and the Brussels Capital Region (Federal states of the Kingdom of Belgium). Florabank covers an area of 13.682 km². Flanders has a temperate maritime climate influenced by the North Sea and the Atlantic Ocean, with relatively moderate summers and mild winters. Flanders is the northern part of Belgium. The two main geographical regions of Flanders are the Yser basin, in the North-West and the central plain. Flanders is divided in 6 ecoregions (Dunes district; Kempens district; Loam district; River Maas; Polder district, Sand and Loam district). The Brussels Capital region is a small region (162 km²) surrounded Flanders and is entirely situated in the Loam district. The majority of this region is highly urbanized and only the southern part is occupied by a large beech forest.

**Coordinates:** 50°37'12"N and 51°29'24"N Latitude; 2°31'12"E and 6°12'0"E Longitude

## Temporal coverage

1800–2011.

### Sampling Methods

**Figure 2.**
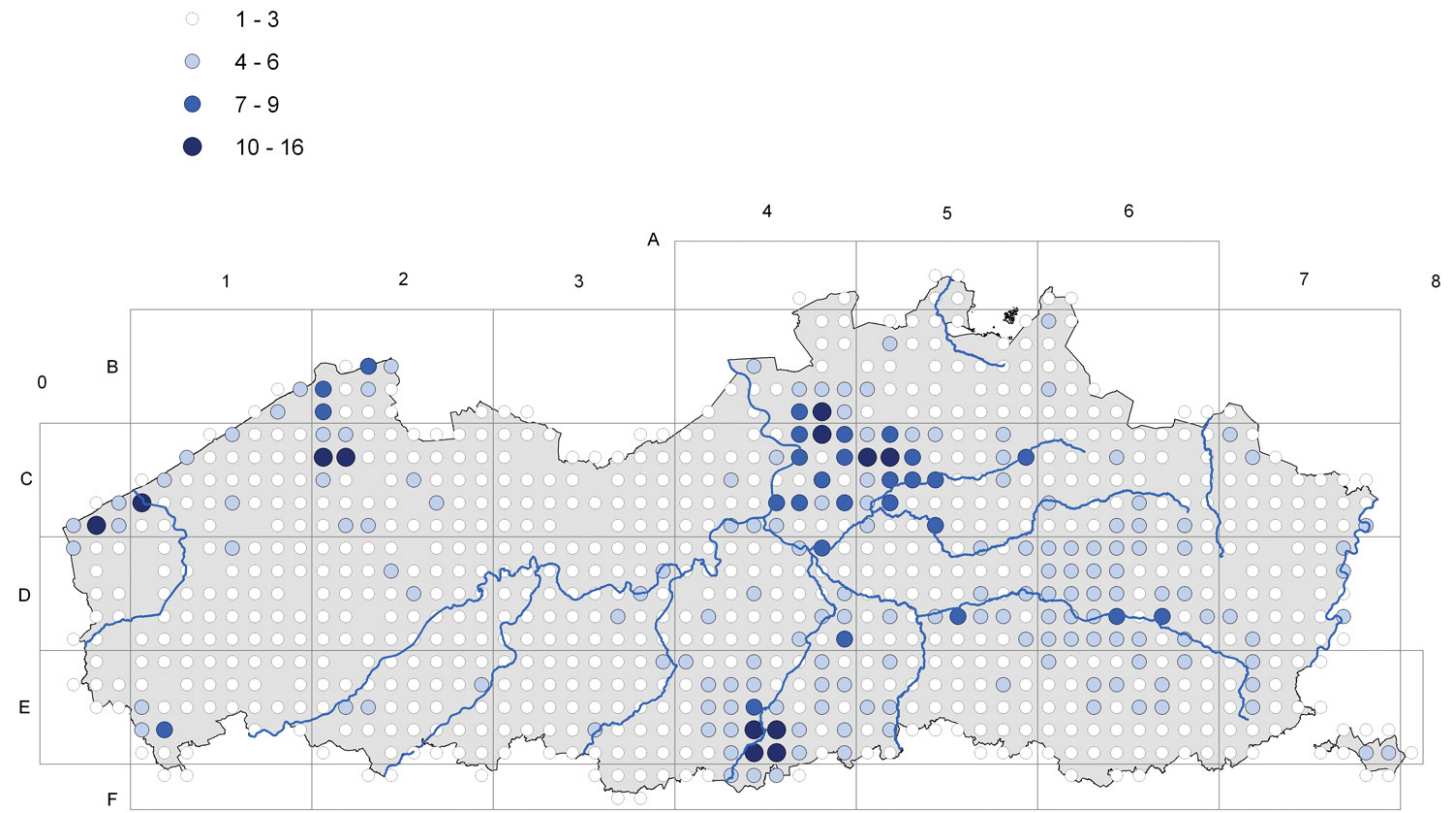
Number of prospected 1 km² grids in each grid of 4×4 km for the period 1939–1971. A 1 km² grid cell is considered as prospected if at least 90 species have been recorded.

**Figure 3.**
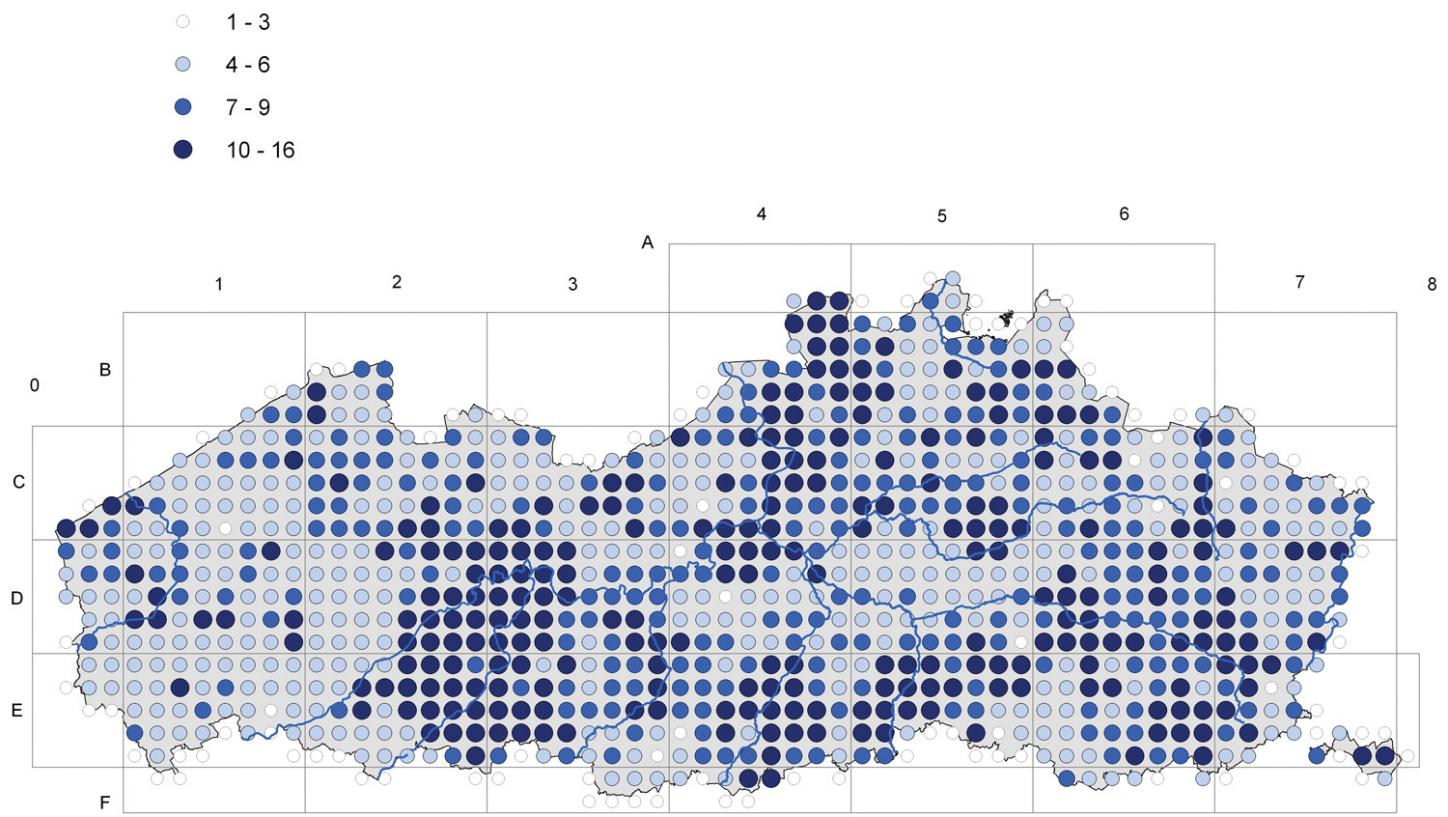
Number of prospected 1 km² grids in each grid of 4×4 km for the period 1972-2004. A 1 km² grid cell is considered as prospected if at least 90 species have been recorded.

The spatial coverage of the territory has evolved through time. The data from the period before 1939 pertains mostly to herbarium specimens and reflects only part of the vascular plant composition of the region. From 1939 onwards most observations are collected using a standardized protocol based on the methodology used for the Atlas of the flora of Belgium and Luxemburg (Van Rompaey and Delvosalle 1972). The atlas area is covered by a grid of 4 × 4 Km squares, which is further subdivided into 1 × 1 Km squares. All species observed during a visit to a grid cell of 1 km² were recorded without distinguishing between common or rare species. In each 4 × 4 km square, more than one 1 km² squares were surveyed. The inventories dating from the period 1939–1971 fed the Atlas of the flora of Belgium and Luxemburg (Van Rompaey and Delvosalle 1972) (figure 2), while those from 1972–2004 served to produce that by Van Landuyt et al. (2006) (figure 3). During the first period (figure 2) only one survey of 1km² in each grid of 4 × 4km was required, during the second period we attempted to obtain data from at least four 1 km² grids in each grid of 4x4 km. From 2005 onwards we continued to gather data using the same protocol.

### Quality Control

All records are validated before they are added to Florabank. The basic reference for quality control is the Belgian atlas (Van Rompaey and Delvosalle 1972). New data to be entered into the database are first submitted to a preliminary, automatic control. Observations pertaining to common species which were previously validated to occur in the neighbouring grid cells of 4 × 4 km squares over the last 35 years are automatically validated. Observations of species that are considered rare, or common species that have not been recorded in the neighbouring grid cells since 35 years are subjected to a manual control by experts. If the record concerns a location validated by other sources (e.g. recent herbarium specimens, peer reviewed papers) it is validated by the managers of the database, if not the observers can be asked to provide extra proof of their observation (e.g. herbarium specimens or photographs). Once an observation is validated (automatically or by the database manager) it can be considered for the validation of new observations.

## Datasets

### Dataset description

The Florabank1 dataset is a custom made SQL view of the Florabank database hosted in the Research Institute for Nature and Forest. The view shows only those data that are accepted for publication in the Darwin Core standard. Fields given are: occurenceID, modified, language, institutionCode, collectionCode, basisOfRecord, catalogNumber, recordedBy, occurrenceDetails, eventDate, country, verbatimLocality, verbatimCoordinates, verbatimCoordinatesystem, decimalLatitude, decimalLongitude, geodeticDatum, coordinateUncertaintyInMeters, scientificName, originalNameUsage, taxonRank, verbatimTaxonrank and nomenclaturalCode.

**Object name:** Darwin Core Archive Florabank1: a grid-based database on vascular plant distribution in the northern part of Belgium

**Character encoding:** UTF-8

**Format name:** Darwin Core Archive format

**Format version:** 1.0

**Distribution:** http://ipt.inbo.be/archive.do?r=florabank1

**Publication date of data:** 2011-03-25

**Language:** Dutch

**Licenses of use:** This work is licensed under a Creative Commons Attribution-NonCommercial-NoDerivs 3.0 Unported License.

**Metadata language:** English

**Date of metadata creation:** 2011-03-25

**Hierarchy level:** Dataset
